# Therapeutics

**Published:** 1880-06

**Authors:** 


### THERAPEUTICS.

THE TOPICAL USES OF ERGOT.—Dr. W. C. Dabney, in an article in the *American Journal of the Medical Sciences*, July, 1879, calls attention to the local use of ergot in various affections. In chronic conjunctivitis, in which the vessels are enlarged and tortuous, he advises the frequent cleansing of the eye with warm water, and the instillation after each washing of a few drops of the following solution: Ergot (solid extract) gr. x, glycerin ʒi; water to make ℥i. This treatment is less applicable in cases in which there is much pain or intolerance of light. In pterygium the same solution may be used with advantage. In cases of pharyngitis, when the vessels are enlarged and tortuous and there is not much secretion, and in hypertrophy of the tonsils the following solution should be painted on the parts twice a day: Ergotin, gr. xx; tincture of iodine, ʒi; glycerin to make ℥i. In cases of cervical metritis, ergot and belladonna may be combined in the following proportions to form pessaries: Ergotin (or solid extract of ergot), gr. xx; extract of belladonna, gr. ii; cocoa butter, q. s., mix and make into six pessaries; one to be inserted into the vagina every night, after using the hot douche. In warm weather these remedies may be dissolved in glycerin and water, as in this formula: Ergotin, gr. iii; extract of belladonna, gr. vi; water and glycerin, āā ℥iv; mix. A pledget of cotton is to be saturated with this solution, and inserted into the vagina at bed-time, after the hot douche; the cotton should be removed in the morning. Dr. S. Eldridge mentions in the *New York Medical Journal*, October, 1879, several other affections in which the local application of ergot is beneficial. He treated an obstinate case of acne rosacea, occurring on the nose of a young lady, by the use of ergotin, applied during the night upon lint; in three weeks there was much improvement, and in six months no trace of the disease was visible. In another case of the same affection, due to drinking, he injected two or three minims of the following preparation of ergotin into the substance of the skin at intervals of three days, having first softened the tissues as much as possible by several days' continuous poulticing: Ergotin, gr. xv; glycerin, gr. xxx; water, ʒii; thoroughly triturate and strain. No suppuration occurred. Thirty injections were made, and in four months the nose was almost natural in appearance. Dr. Eldridge also gives details of some cases of gonorrhœa and granular urethritis which he has treated by ergotin locally, with marked success. The remedy may be introduced into the urethra either by means of the ointment syringe or rubbed into the meshes of a cylindrical hollow lamp-wick, which is supported by a small bougie passed into its center, this swab being allowed to remain in the urethra for about half an hour. In an old standing case of otitis media, accompanied by destruction of the membrana tympani, large granulations and profuse discharge, ergotin was applied directly with a camel's hair pencil, after having been diluted with sufficient glycerin to make it flow easily; the result was satisfactory, the granulations having shrunk rapidly, while the discharge disappeared and the sensitiveness abated. The author also suggests that in eczema, vaginal leucorrhœa and nasal catarrh the topical application of ergotin should prove of value.—*The London Medical Record*, January 15, 1880.

